# Real-Time EEG-Derived Amygdala Neurofeedback for Post-Traumatic Stress Disorder: A Clinical Case Series

**DOI:** 10.3390/jcm15062122

**Published:** 2026-03-11

**Authors:** Diana Ghelber, Tal Harmelech, Aron Tendler

**Affiliations:** 1 Institute of Advanced Psychiatry, Fort Worth, TX 76132, USA; dghelber95@gmail.com; 2 GrayMatters Health, Haifa 3303403, Israel; tal.harmelech@gmail.com

**Keywords:** PTSD, neurofeedback, EEG-fMRI, amygdala, real-world evidence, self-regulation, self-neuromodulation, community practice, PCL-5

## Abstract

**Background:** Post-traumatic stress disorder (PTSD) affects millions globally, with 40–50% of patients not responding adequately to first-line treatments. Prism neurofeedback, an FDA-cleared electroencephalography (EEG)-based system targeting amygdala-derived biomarkers, has demonstrated efficacy in randomized controlled trials (RCTs) and multicenter studies. Real-world implementation data from community clinical practice remain limited. **Objective:** To evaluate clinical outcomes and patient-developed self-regulation strategies of Prism neurofeedback in patients with PTSD in community clinical practice. **Methods:** Retrospective case series of 28 consecutive patients with PTSD treated with Prism neurofeedback in a community psychiatry practice. The primary outcome was change in PTSD Checklist for DSM-5 (PCL-5) from baseline to end of treatment. **Results:** Twenty-one of 28 patients (75.0%) completed treatment. Mean PCL-5 reduction was 37.0 ± 18.2 points (Cohen’s d = 2.03). Response rates were 100% for any improvement and 90.5% for clinically significant improvement (≥10-point reduction). Five patients (23.8%) achieved excellent response with ≥50-point reduction. Limited follow-up data (1–3 months post-treatment) were available for three patients; two of three (67%) exceeded their end-of-treatment gains. Four patients receiving booster sessions showed continued improvement. **Limitations:** The uncontrolled, retrospective design precludes causal attribution of improvements to the intervention versus placebo effects or regression to the mean. The 25% early discontinuation rate may introduce attrition bias. Durability data are available for only three patients. **Conclusions:** This case series provides real-world evidence supporting the feasibility and potential clinical utility of Prism neurofeedback in community practice, with outcomes comparable to controlled studies and preliminary evidence of durable treatment effects. These findings complement existing RCT evidence by demonstrating successful implementation outside research settings.

## 1. Introduction

Post-traumatic stress disorder affects approximately 7% of the population and represents one of the most challenging mental health conditions in clinical practice (DSM-5-TR). Despite advances in evidence-based treatments, 40–50% of patients do not achieve adequate symptom remission. Trauma-focused psychotherapies typically achieve response rates of 50–60% [1], while pharmacotherapy shows modest efficacy often accompanied by problematic side effects [2]. This persistent treatment gap underscores the need for novel, neurobiologically informed interventions that can complement or extend existing approaches.

Central to PTSD pathophysiology is amygdala hyperactivity coupled with impaired fear extinction and prefrontal cortex hypoactivation [3]. These circuit-level abnormalities represent a compelling target for direct neural intervention. Neurofeedback approaches that enable patients to learn real-time self-neuromodulation of amygdala activity offer a potential mechanism to address these underlying dysfunctions without pharmacological side effects and may enhance the neural substrates engaged by trauma-focused psychotherapy.

The Prism neurofeedback system is an FDA-cleared intervention indicated as an adjunctive treatment for PTSD, designed to complement existing therapeutic approaches either as monotherapy or in combination with other treatments. The system utilizes an EEG-based approach targeting amygdala-derived biomarkers through real-time feedback, employing a sophisticated amygdala-derived EEG-functional magnetic resonance imaging (fMRI) Pattern (EFP) biomarker developed through machine learning models trained on simultaneous EEG-fMRI data [4]. An interactive audiovisual interface with animated virtual characters responding dynamically to brain activity enables patients to develop personalized self-regulation strategies [5].

Controlled trials have established the efficacy of this approach. Keynan and colleagues [6] showed significant improvements in stress resilience and PTSD symptoms in a randomized controlled trial. Fruchter and colleagues [7] reported a 67% CAPS-5 response rate in a multicenter trial of 79 participants. Goldental et al. [8] reported significant improvements across PTSD symptom clusters with associated emotional regulation changes. Fine et al. [9] demonstrated feasibility and preliminary efficacy in treatment-resistant childhood sexual abuse PTSD. Voigt and colleagues [10] provided meta-analytic support across multiple randomized controlled trials.

While controlled studies have established efficacy under research conditions, real-world implementation data from routine clinical practice remain limited. Community psychiatric settings differ meaningfully from clinical trial environments in clinical complexity, comorbidity burden, scheduling constraints, and clinician variability—factors that may influence both feasibility and outcomes. Translational data from community settings are therefore essential for informing clinical decision making and evaluating whether trial-demonstrated benefits generalize to practice. The present study examines Prism neurofeedback implementation and outcomes in a community psychiatry practice.

## 2. Methods

### 2.1. Study Design and Ethics

This retrospective case series was conducted in a community psychiatry practice in Fort Worth, TX, USA. Ethical approval was obtained through Sterling IRB with a waiver of informed consent (Protocol CLP500, 18 March 2025), with strict HIPAA compliance maintained.

### 2.2. Participants

Inclusion criteria required DSM-5 PTSD diagnosis confirmed through clinical interview, age 18–75 years, and capacity to provide informed consent. Exclusion criteria encompassed EEG contraindications, active psychosis or mania, severe substance use disorder requiring immediate intervention, and cognitive impairment precluding participation.

The patient sample reflected substantial clinical complexity and treatment resistance (aged 21–69 years, including healthcare professionals and individuals with advanced degrees). Several patients had failed multiple prior treatments including transcranial magnetic stimulation (TMS), ketamine therapy, intensive outpatient programs (IOP), and extended psychotherapy. Comorbidities included traumatic brain injury (TBI), bipolar disorder, OCD, chronic pain, and ongoing medical trauma.

Of the 21 patients in the final analysis, 18 received Prism as monotherapy while 3 (14.3%) received it as adjunctive treatment concurrent with other interventions: one with TMS and two with ketamine therapy. This reflects Prism’s FDA-cleared indication as an adjunctive treatment with no contraindications for concurrent use.

### 2.3. Prism Neurofeedback Intervention

The Prism system employs a wireless, non-invasive EEG headset that continuously records scalp electrical activity across multiple channels. This signal is processed in real time using a proprietary amygdala-derived EEG-fMRI Pattern (EFP) biomarker, developed through machine learning models trained on simultaneous EEG-fMRI recordings from healthy volunteers. The EFP provides a validated real-time index of right amygdala BOLD activity derived from surface EEG alone, without requiring concurrent neuroimaging [4].

Patients view an interactive audiovisual display in which animated virtual characters respond dynamically to moment-to-moment changes in their EFP signal—providing intuitive feedback about their amygdala regulation state. This enables patients to identify and refine personalized mental strategies (e.g., imagery, memory recall, focused attention or sensory anchoring) that reliably downregulate amygdala activity. Each session lasted approximately an hour, comprising fifteen minutes of EEG setup and planning, thirty minutes of active neurofeedback, and fifteen minutes of debriefing. The process-based framework underlying this approach is described in detail elsewhere [5].

Sessions were conducted in-clinic and scheduled at a frequency of approximately two to three per week. Treatment consisted of up to 16 sessions based on the standard Prism protocol, individualized according to clinical response and patient availability.

### 2.4. Outcome Measures

The primary outcome was the change in PTSD Checklist for DSM-5 (PCL-5) score from baseline to end of treatment. The PCL-5 is a validated 20-item assessment with scores ranging from 0–80 points [11]. Baseline assessments were completed prior to the first Prism session, at the initial intake or setup appointment, prior to any neurofeedback. Subsequent assessments were conducted approximately every 2–4 sessions, administered at the start of each assessment visit before that day’s session. Change scores were calculated as endpoint minus baseline with negative values indicating symptom reduction.

Response criteria were defined as: any improvement (>0-point reduction), clinically significant improvement (≥10-point reduction; [12]), conservative threshold (≥15-point reduction), large improvement (≥30-point reduction), and excellent response (≥50-point reduction). Treatment completers were defined as patients who completed an endpoint PCL-5 assessment, regardless of the number of sessions attended; patients who discontinued within the first four sessions without an endpoint assessment were classified as non-completers. For patients with follow-up assessments (1–3 months post-treatment), data were analyzed separately to evaluate response durability. Patient-developed self-regulation strategies, recorded in clinical session notes, were extracted retrospectively and classified by content type.

### 2.5. Statistical Analysis

Analysis employed paired *t*-tests for baseline to endpoint PCL-5 changes and Cohen’s d effect size calculations [13]. Response rates were calculated as proportions with 95% confidence intervals using the Wilson score method [14].

## 3. Results

### 3.1. Treatment Implementation

**Figure 1:**
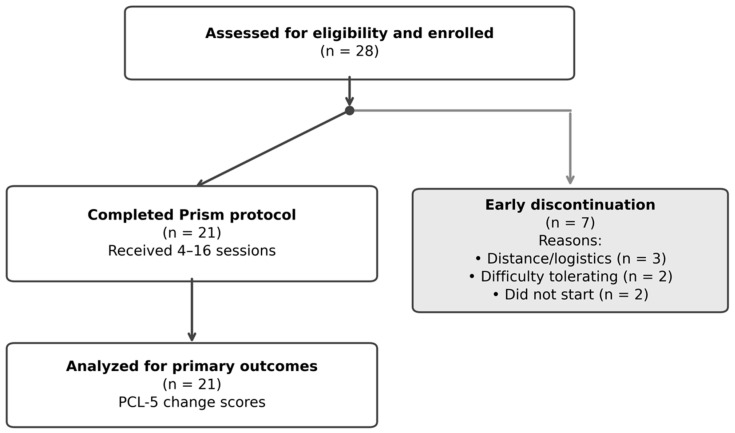
CONSORT diagram. All enrolled participants received baseline assessment. Early discontinuation defined as withdrawal within first 4 sessions. No participants excluded from analysis.

Twenty-eight consecutive patients enrolled; 21 (75.0%) completed treatment and 7 (25%) discontinued within the first four sessions (Figure 1, Table 1). Reasons for discontinuation included distance/logistical barriers (*n* = 3), difficulty tolerating the intervention (*n* = 2), and failure to complete enrollment (*n* = 2). Non-completers had comparable baseline PCL-5 scores to completers (mean 53.8 vs. 56.9). Completed sessions ranged from 4–16 (mean: 13.0 ± 3.8) over 3–10 weeks. No serious adverse events, medical complications, or psychiatric exacerbations requiring intervention were observed.

### 3.2. Primary Outcome: PTSD Symptom Changes

**Figure 2:**
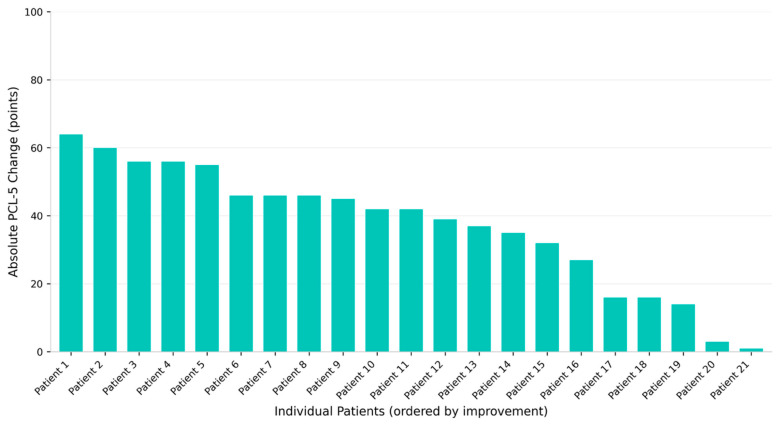
Individual patient changes in PCL-5 scores from baseline to endpoint, ordered by magnitude of improvement. Each bar represents one patient (*n* = 21). Positive changes indicate symptom reduction. All patients (100%) showed improvement, with 19 patients (90.5%) achieving clinically significant improvement (≥10-point reduction). Patient identifiers are anonymized.

Mean baseline PCL-5 was 56.9 ± 14.1 points (range: 21–73; Table 2, Figure 2). At end of treatment, scores decreased to 19.8 ± 17.8 points (range: 1–69), representing a mean reduction of 37.0 ± 18.2 points—a 65.2% average improvement with a large effect size (Cohen’s d = 2.03). This improvement was highly significant (*p* < 0.001, paired *t*-test; 95% CI: 28.7–45.4 points).

Response rates were: any improvement 100% (21/21; 95% CI: 84.5–100%), clinically significant improvement 90.5% (19/21; 95% CI: 71.1–97.3%), and conservative threshold 85.7% (18/21; 95% CI: 65.4–95.0; Table 3). Fifteen patients (71.4%) achieved large improvement (≥30-point reduction), and five patients (23.8%) achieved excellent response (≥50-point reduction).

### 3.3. Treatment Follow-Up

Follow-up data (1–3 months) were available for three patients. Two of three (67%) continued to improve beyond end-of-treatment scores, with mean additional improvement of 5 points (range: 4–6). One patient initially improved but experienced symptom recurrence at 3-month follow-up (PCL-5: 8 → 0 → 14). Given the small number of patients with follow-up data, these observations are descriptive and should not be taken as definitive evidence of durability.

Four patients received booster sessions (1–3 sessions) after completing initial treatment and demonstrated continued symptom reduction, with mean additional improvement of 19.3 points (range: 11–33). These findings suggest that self-regulation skills may continue to develop with ongoing practice, though larger prospective studies are needed to characterize long-term outcomes.

### 3.4. Subgroup Analyses

**Figure 3:**
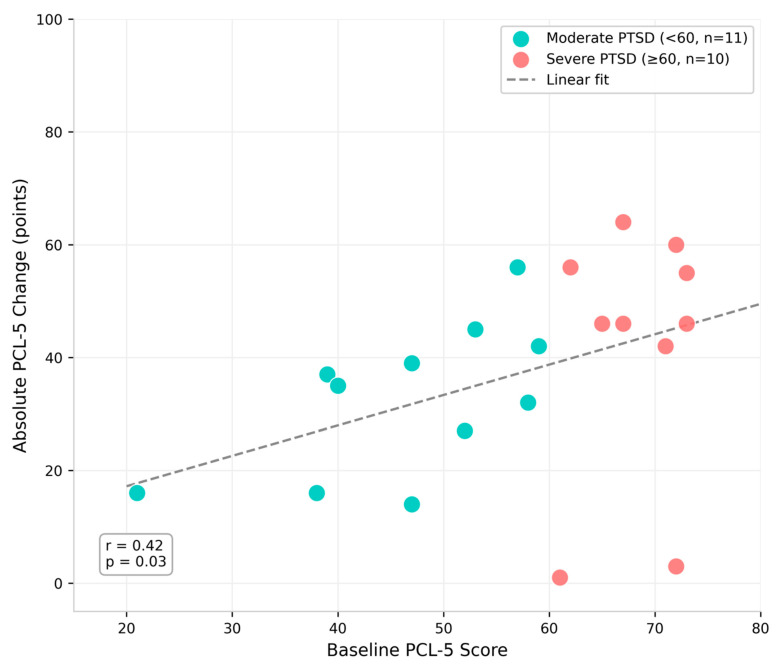
Relationship between baseline PCL-5 severity and absolute symptom change. Each point represents one patient (*n* = 21). Patients with severe baseline PTSD (≥60, pink circles) showed greater absolute improvements than those with moderate PTSD (<60, cyan circles). The correlation (r = 0.42, *p* = 0.03) may reflect regression to the mean rather than differential treatment response.

Patients with severe baseline PTSD (PCL-5 ≥ 60, *n* = 10) showed numerically greater absolute improvements than those with moderate symptoms (PCL-5 < 60; *n* = 11; Table 4, Figure 3). A moderate correlation was observed between baseline severity and symptom change magnitude across the full sample (r = 0.42, *p* = 0.03). This pattern likely reflects greater room for improvement at higher baselines—consistent with regression to the mean—rather than differential treatment efficacy.

Patients completing ≥12 sessions demonstrated numerically superior outcomes, and early discontinuation patterns suggest initial engagement may predict treatment completion.

### 3.5. Exploratory Self-Regulation Strategy Analysis

Strategy documentation was available for 11 of 21 patients (52.4%). Among patients with available data, 1–9 distinct strategies were documented per patient (mean: 4.1 ± 2.8) across seven categories (Table 5 and Table S1): visualization/imagery, auditory/musical, sensory/physical, memory-based, animal/riding, nature/environmental, and abstract/emotional.

**Figure 4:**
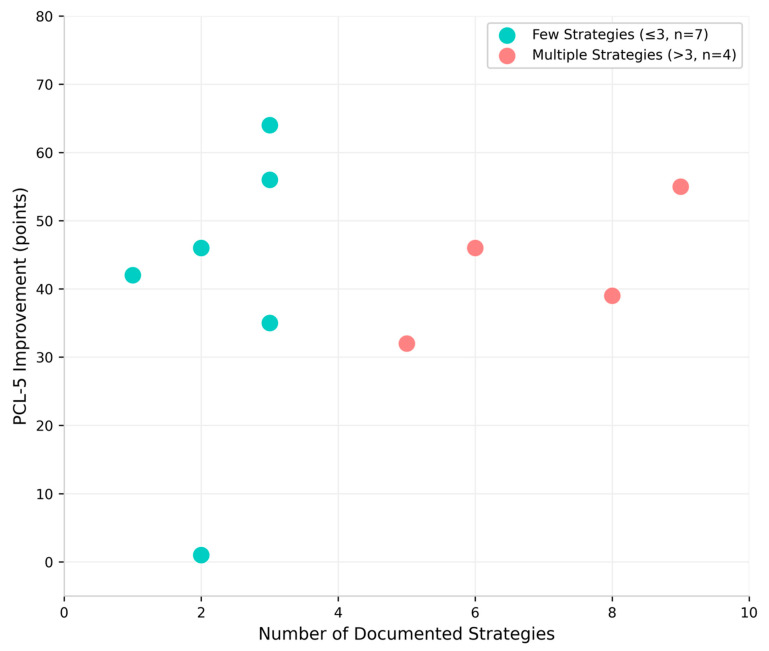
Relationship between number of documented self-regulation strategies and PCL-5 improvement among patients with strategy data (*n* = 11). Patients with multiple strategies (>3, *n* = 4, pink circles) and few strategies (≤3, *n* = 7, cyan circles) achieved nearly identical improvements (43.0 vs. 42.9 points), demonstrating that strategy quantity does not predict outcome (r = 0.18, *p* = 0.59).

Patients developing multiple strategies (>3, *n* = 4) achieved mean PCL-5 reduction of 43.0 ± 9.8 points versus 42.9 ± 20.9 points for those with fewer strategies (*n* ≤ 3, *n* = 7; difference = 0.1 points, Cohen’s d = 0.01, *p* = 0.99; Figure 4, Table S2). No significant correlation was found between strategy count and outcomes (r = 0.18, *p* = 0.59). These analyses are exploratory given the incomplete and retrospective nature of strategy documentation and should be considered hypothesis-generating. These findings suggest that strategy quantity does not predict treatment outcomes, as patients with multiple versus few strategies achieved virtually identical improvements. Rather, quality of strategy engagement and degree of personalization may be more important than the number of distinct strategies documented.

### 3.6. Individual Patient Outcomes

Several patients achieved exceptional response (≥50-point reductions). One with military PTSD and post-TBI achieved a 64-point reduction (67 to 3), another with childhood trauma showed a 60-point reduction (72 to 12), and two achieved a 56-point reduction (57 to 1; 62 to 6), including one with military and childhood trauma plus TBI.

Among patients receiving adjunctive treatment, outcomes were mixed. One patient receiving concurrent TMS achieved a 27-point reduction (52 to 25), while another receiving ketamine therapy showed a 46-point improvement (73 to 27). However, one patient receiving ketamine showed minimal response (3-point reduction: 72 to 69).

One patient showed poor response, achieving minimal improvement (1-point reduction: 61 to 60), highlighting individual variability in treatment response (Table 6).

## 4. Discussion

### 4.1. Principal Findings

This case series demonstrates that Prism neurofeedback can be successfully implemented in community practice, with 75.0% treatment completion and a large effect size (Cohen’s d = 2.03). The clinically significant improvement rate of 85.7% compares favorably to established PTSD treatments [2] and exceeds the 67% CAPS-5 response rate reported in the multicenter controlled trial by Fruchter and colleagues [7]. The mean PCL-5 reduction of 37.0 points represents substantial clinical improvement, with effect sizes within the range reported for strongly recommended psychotherapies [15].

Notably, the sample included patients with considerable clinical complexity—multiple treatment failures, TBI, bipolar disorder, chronic pain, and ongoing medical trauma—who would often be excluded from or underrepresented in controlled trials. The consistency between these real-world outcomes and controlled trial results therefore strengthens confidence in the external validity of the RCT evidence base and suggests Prism neurofeedback may be robust to the heterogeneity encountered in community practice.

The large uncontrolled effect size should nonetheless be interpreted in the context of an open-label case series. Improvements may partly reflect placebo effects, regression to the mean (particularly in patients with high baseline severity), natural symptom fluctuations, or other uncontrolled factors. These considerations do not diminish the translational value of the findings, but they do underscore the need for controlled trials in community settings to establish causal efficacy.

### 4.2. Adjunctive Use and Bridge Utility

Prism demonstrated effectiveness both as monotherapy (*n* = 18) and as adjunctive treatment (*n* = 3), consistent with its FDA clearance for flexible clinical integration. This demonstrates real-world utility across treatment contexts, with no observed contraindications for concurrent use with other evidence-based therapies.

Several cases demonstrated Prism’s utility as a bridge intervention. One patient with severe obstructive sleep apnea and avoidance completed a sleep study and accepted continuous positive airway pressure therapy after anxiety reduction. Another resumed psychotherapy after Prism improved treatment readiness. A third patient at high suicide risk avoided hospitalization and engaged with IOP after combined TMS, ketamine, and Prism treatment. These patterns suggest that symptom reduction with Prism may enhance patients’ capacity to engage with other evidence-based treatments, a hypothesis warranting systematic prospective evaluation.

### 4.3. Treatment Mechanisms

The diversity of patient-developed strategies—from visualization to musical to memory-based approaches—suggests the intervention may support amygdala self-regulation through multiple pathways. Strategy count did not predict outcomes, with patients developing multiple (>3) versus few (≤3) strategies achieving virtually identical improvements (43.0 vs. 42.9 points, Cohen’s d = 0.01, *p* = 0.99). This suggests that the depth and personalization of strategy engagement may matter more than the number of distinct strategies documented. These findings are exploratory given the partial and retrospective nature of strategy documentation, but they provide a foundation for prospective investigation of the mechanisms through which patients learn amygdala self-regulation.

### 4.4. Limitations

The retrospective, uncontrolled design prevents definitive attribution of improvements to the intervention versus placebo effects or regression to the mean. Single-center design limits generalizability, and small sample size constrains subgroup analyses. The 25% early discontinuation rate, primarily due to practical barriers, may introduce some attrition bias, though comparable baseline scores between completers and non-completers partially mitigate this concern. Follow-up data were available for only three patients, limiting conclusions about response durability. Strategy documentation was incomplete, capturing only a subset of patient experiences. Future research should include RCTs in community settings, systematic prospective follow-up, and structured documentation of strategy development. The three patients receiving adjunctive treatments (TMS or ketamine) are too few to permit any comparison with the monotherapy group, and outcomes in these patients cannot be attributed to Prism specifically.

## 5. Conclusions

This case series provides real-world evidence supporting the feasibility and potential clinical utility of Prism neurofeedback for PTSD treatment in community practice. With 85.7% achieving clinically significant improvement and a large effect size (Cohen’s d = 2.03), outcomes were comparable to controlled studies. The 75.0% completion rate demonstrates realistic implementation expectations. These findings complement existing randomized controlled trial evidence by demonstrating successful implementation outside research settings for this FDA-cleared adjunctive treatment. Prospective controlled trials in community settings are needed to confirm causal efficacy and characterize long-term outcomes.

## Figures and Tables

**Table 1:** Treatment Completion and Feasibility (*n* = 28).

Characteristic	Value
Total Enrolled	28 patients
Treatment Completers	21 (75.0%)
Early Discontinuation	7 (25.0%)
Sessions Completed (mean ± SD)	13.0 ± 3.8
Session Range	4–16 sessions
Treatment Duration	3–10 weeks
Adverse Events	None documented

**Table 2:** Primary Outcome Summary Statistics (*n* = 21).

Measure	Value	95% Confidence Interval
Baseline PCL-5	56.9 ± 14.1	50.4–63.3
Endpoint PCL-5	19.8 ± 17.8	11.7–27.9
Mean Change	−37.0 ± 18.2	28.7–45.4
Percent Improvement	65.2%	-
Effect Size (Cohen’s d)	2.03	-
*p*-value	<0.001	-

**Table 3:** Response rates by improvement threshold (*n* = 21).

Response Threshold	Responders	Response Rate	95% CI
Any improvement (>0 points)	21/21	100%	84.5–100%
Clinically significant (≥10 points)	19/21	90.5%	71.1–97.3%
Conservative threshold (≥15 points)	18/21	85.7%	65.4–95.0%
Large improvement (≥30 points)	15/21	71.4%	50.0–86.2%
Excellent response (≥50 points)	5/21	23.8%	9.6–45.1%

**Table 4:** Baseline severity subgroup analysis (*n* = 21).

Characteristic	Severe PTSD (≥60)	Moderate PTSD (<60)
*n*	10	11
Baseline PCL-5	68.3 ± 4.5	46.5 ± 11.4
Endpoint PCL-5	26.4 ± 21.8	13.8 ± 11.2
Mean Change	−41.9 ± 22.2	−32.6 ± 13.4
Sessions Completed	14.2 ± 2.2	11.9 ± 4.7
Any improvement	10 (100%)	11 (100%)
Clinically significant	8 (80%)	11 (100%)
Conservative threshold	8 (80%)	10 (90.9%)

**Table 5:** Strategy categories and clinical examples (*n* = 11 patients, 40 strategy instances).

Strategy Category	Count	Percentage	Representative Examples
Abstract/Emotional	10	25.0%	“360 Acceptance”, “sense of freedom”, “promise of life”
Sensory/Physical	9	22.5%	“touching pants and focusing on feet”, “breathing”, “mindful touch”
Visualization/Imagery	5	12.5%	“creative tension”, “seed zone”, “spaced out into Mars”
Nature/Environmental	5	12.5%	“sound of wind and birds”, “safety when in nature”, “bird song”
Auditory/Musical	5	12.5%	“humming a song”, “music”, “songs”
Animal/Riding	3	7.5%	“riding her pony”, “riding her big horse”, “riding shorty”
Memory-Based	3	7.5%	Christmas memories”, “wifey and catch”, “family/England/age 5”

Note: Patients often developed multiple strategies across different categories.

**Table 6:** Individual patient PCL-5 outcomes (*n* = 21).

Patient	Baseline PCL-5	Endpoint PCL-5	Change	% Change	Sessions	Response Category
1	67	3	−64	−96%	15	Excellent (≥50-point)
2	72	12	−60	−83%	14	Excellent (≥50-point)
3	57	1	−56	−98%	9	Excellent (≥50-point)
4	62	6	−56	−90%	15	Excellent (≥50-point)
5	73	18	−55	−75%	15	Excellent (≥50-point)
6	67	21	−46	−69%	15	Good (30–49-point)
7	73	27	−46	−63%	15	Good (30–49-point)
8	65	19	−46	−71%	8	Good (30–49-point)
9	53	8	−45	−85%	15	Good (30–49-point)
10	59	17	−42	−71%	16	Good (30–49-point)
11	71	29	−42	−59%	15	Good (30–49-point)
12	47	8	−39	−83%	15	Good (30–49-point)
13	39	2	−37	−95%	7	Good (30–49-point)
14	40	5	−35	−88%	15	Good (30–49-point)
15	58	26	−32	−55%	15	Good (30–49-point)
16	52	25	−27	−52%	15	Moderate (10–29-point)
17	21	5	−16	−76%	5	Moderate (10–29-point)
18	38	22	−16	−42%	15	Moderate (10–29-point)
19	47	33	−14	−30%	4	Moderate (10–29-point)
20	72	69	−3	−4%	15	Minimal (<10-point)
21	61	60	−1	−2%	15	Minimal (<10-point)

Note: Response categories based on absolute PCL-5 point changes.

## Data Availability

All the data are included in the manuscript.

## Supplementary Materials

The following supporting information can be downloaded at: https://www.mdpi.com/article/10.3390/jcm15062122/s1, Table S1: Individual Patient Strategy Development Profiles; Table S2: Multiple Strategy Development Analysis.
