# Supplementary material for: Real-Time EEG-Derived Amygdala Neurofeedback for Post-Traumatic Stress Disorder: A Clinical Case Series
Source: J Clin Med. 2026 Mar 11;15(6):2122. doi: 10.3390/jcm15062122 (PMC13026192; doi:10.3390/jcm15062122)
Supplement: Supplementary file 1 [file jcm-15-02122-s001.zip › jcm-4166912-supplementary.pdf]

Supplementary Tables

Table S1. Individual Patient Strategy Development Profiles

Patient	PCL-5 Change	Strategy Count	Primary Categories	Representative Strategies
Patient 5	-55	9	Visualization, Nature, Abstract	"Creative Tension", "Seed zone", "360 Acceptance", “promise of life”, “wind and birds”
Patient 12	-39	8	Auditory, Animal/Riding, Abstract	"humming a song", "riding her pony", "sense of freedom"
Patient 6	-46	6	Sensory, Auditory, Abstract	"touching pants/feet”, “music”, “creating fashion”
Patient 15	-32	5	Visualization, Nature, Sensory	"large bird with wings”, “being out in nature”, “swimming in lake”
Patient 1	-64	3	Memory	"honeymoon”, “Disneyland”, “trip to Alaska”
Patient 4	-56	3	Memory, Abstract, Auditory	“wifey and catch”, “feeling on stage”, “music”
Patient 14	-35	3	Abstract, Visual	"Morning quiet time”, “Morning snuggles”, “focus on yellow sign”
Patient 7	-46	2	Auditory, Sensory	“songs”, “breathing”
Patient 21	-1	2	Auditory, Abstract	“singing”, “un-wrinkling brain”
Patient 3	-56	1	Memory	"family/England/age 5"
Patient 11	-42	1	Visualization	"spaced out into Mars"

Note: Only patients with documented strategy data (n=11)

Table S2. Multiple Strategy Development Analysis

Strategy Development	n	Strategies per patient	Mean PCL-5 Change (SD)	Effect Size
Multiple strategies (>3)	4	Range 5–9	43.0 ± 9.8	-

Strategy Development	n	Strategies per patient	Mean PCL-5 Change (SD)	Effect Size
Few strategies (≤3)	7	Range 1–3	42.9 ± 20.9	-
Difference	-		0.1	Cohen's d = 0.01

Note: Ranges refer to the number of documented self-regulation strategies per patient (minimum = 1, maximum = 9 in this sample). Exploratory analysis with small sample size. Pattern shows no clear advantage for multiple strategy development.
